# Editorial: Technologies to support elderly patients with dementia

**DOI:** 10.3389/fnagi.2023.1333724

**Published:** 2023-11-27

**Authors:** Luigi Bibbo, Francesco Carlo Morabito, Fabio La Foresta, Alessia Bramanti

**Affiliations:** ^1^Biorobotic Lab, Department of Industrial Engineering, University of Florence, Florence, Italy; ^2^DICEAM, Mediterranea University of Reggio Calabria, Reggio Calabria, Italy; ^3^Department of Medicine, Surgery and Dentistry, University of Salerno, Baronissi, Italy

**Keywords:** artificial intelligence, IoT, mild cognitive impairment, virtual reality, dementia—Alzheimer's disease

Advances in medicine and rising living standards contribute to increased life expectancy and, consequently, to the growth of people with dementia (Alzheimer's Association, 2018). The precise causes of the development of dementia are not fully understood. Various degenerative, vascular, or traumatic diseases can cause it. Genetic factors and environmental influences are also considered possible causes. The disease initially manifests itself with minor ailments and then develops over time. This stage between the cognitive decline of normal aging and the more severe decline of dementia is known as mild cognitive impairment (MCI). It is characterized by problems with memory, language, and behavior (Knopman and Petersen, 2014). Patients lose their long-term and short-term memory skills. This loss increases dependence, care, and support needs (Yates et al., 2019). It represents one of the most critical problems of our time, both medically and socioeconomically. It produces profound changes in the quality of life of affected families (Aza et al., 2022). Research in recent years has focused on creating various technological solutions based on IoT, Big Data, robotics, and artificial intelligence, both to support patients and caregivers and for doctors for clinical assessments. It produces a substantial reduction in costs and improves access to healthcare. Some of these solutions can monitor various activities and situations via intelligent sensors, while others are oriented toward the evaluation and rehabilitation of patients.

The purpose of this Research Topic is to bring together a collection of papers illustrating an exciting scenario on technological advances, as well as new functionalities and approaches for diagnosis. For example, Lazarou et al. proposed an innovative approach to studying AD-related early cognitive decline by analyzing subjects' brain waves during meditation exercises. The waves were detected with a portable, wireless EEG (MUSE EEG). The use of meditation techniques is a cognitive stimulation practice and a potential AD prevention technique (Wong et al., 2017). In addition, electroencephalography is an effective tool for detecting abnormalities in brain activity in AD. The results discriminated early cognitive decline and brain alterations in different meditation sessions between healthy subjects and those with cognitive impairment.

Virtual reality (VR), a branch of ICT, has been used in dementia and is effective in assessing and screening functional abilities in cognitive impairment. The results showed that it could assist patients and their families by providing memory aids and educational support (García-Betances et al., 2014). Stasolla and Di Gioia proposed a combined system of VR and Reinforcement Learning (RL) as a rehabilitation and evaluation approach for mild cognitive impairment (MNI). It represents a transitional stage between normal cognitive decline due to progressive aging and an early stage of dementia. Experience has shown that people with Mild Cognitive Impairment (MCI) have a higher risk of suffering from dementia than young and older people without MCI because their subjective cognitive decline is unlikely to be identified with an MNI diagnosis (Bradfield, 2023). This combined system allows for early assessment that can be instrumental in the assessment or recovery of cognitive functioning. It has also proven to be effective as a rehabilitation tool; with the AI RL system, the difficulty of the exercises and tasks for the patient can be customized, while with VR technology, an immersive environment similar to real life is created, thus ensuring a continuous and rigorous personalized adaptation to the task or difficulty of the activity.

Social Alarm (SA) is believed to be a technology that allows seniors to live a safe and independent life in their own homes while facing problems such as the risk of accidents due to falls or feelings of insecurity. The SA is an active sensor consisting of a pendant or bracelet worn on the body with a built-in alarm button and a unit connected to the senior's telephone line. Puaschitz et al. surveyed the availability of this system with respect to its use with people with dementia living in their own homes and informal caregivers. The municipalities in Norway offer people over the age of 70 in need of home care free of charge to the SA. The LIVE@Home.Path mixed-method survey collected data from questionnaires and interviews among 82 home-based people with dementia and 278 informal caregivers. The results first showed a gap between access to and use of social alarms, and opinions on their usefulness differ between patients and caregivers. It also emerged the need for follow-up procedures on the installed SA and the verification of the usability of passive sensor technology.

The need to identify individuals at risk of dementia early has stimulated research to develop computerized cognitive tests as alternative to traditional neuropsychological assessments. Na et al. used the computerized cognitive test, the Inbrain cognitive screening test (CST), on a 12-inch tablet PC, and the results obtained were compared with those of the standard neuropsychological test used in Korea, CERAD-K. CST has been previously validated in the following populations: cognitively normal, subjective cognitive decline, mild cognitive impairment (MCI), and dementia (Chin et al., 2020). One hundred sixty-six participants, including 73 cognitively non-impaired individuals and 93 patients with MCI, underwent five different tests. These subtests of the Inbrain CST showed good correlations with each corresponding task of the traditional neuropsychological test, CERAD-K, in patients with MCI and cognitively non-impaired individuals. Therefore, Inbrain CST can be valid in identifying objective cognitive deficits and specific features of cognitive decline with significant advantages over traditional test.

Monitoring the progression of age-related diseases requires accurate methods of brain fragmentation and must account for ethnic differences. Lim et al. developed, for older East Asian individuals, a new comprehensive 3D deep learning model for automatic brain MRI parceling using N-way multiple 3D UNet architectures combined with three memory reduction techniques: inception blocks, dilated convolutions, and attention gates. It shows comparable or better performance than an existing model, FreeSurfer (Fischl et al., 2002). The rapid and robust fragmentation obtained by the model provides crucial data for early detection or monitoring of the progress of neurodegenerative diseases.

Research has produced such results that lead us to believe that technology is not only helpful in the clinical detection of warning signs of cognitive degeneration but can also be used in the maintenance of cognitive health through learning new things and social connections by slowing down the development of the disease. It is therefore foreseeable, in the coming years, the evolution of innovative intelligent models capable of preventing the onset of the disease and the creation of strategies aimed at cognitive stimulation.

## Author contributions

LB: Writing—original draft, Writing—review & editing. FM: Review & editing. FL: Review & editing. AB: Review & editing.
